# Extended use of brentuximab vedotin before autologous stem‐cell transplantation would benefit refractory systemic anaplastic large‐cell lymphoma

**DOI:** 10.1002/ccr3.1461

**Published:** 2018-03-08

**Authors:** Youngil Koh

**Affiliations:** ^1^ Department of Internal Medicine Seoul National University Hospital Seoul 03080 Korea

**Keywords:** Brentuximab vedotin, extended treatment, number of cycles, optimal response, second‐line therapy, systemic anaplastic large‐cell lymphoma

## Abstract

The optimal number of brentuximab vedotin cycles in the treatment of systemic anaplastic large‐cell lymphoma (sALCL) prior to autologous stem‐cell transplantation (ASCT) is unknown. This case illustrates the possible benefit of prolonged brentuximab vedotin before ASCT in sALCL and may help clinical decision‐making, especially in chemorefractory disease.

## Introduction

Brentuximab vedotin (BV; Adcetris^®^) is a CD30‐directed antibody–drug conjugate with notable activity in the treatment of systemic anaplastic large‐cell lymphoma (sALCL). It can be used as salvage chemotherapy prior to autologous stem‐cell transplantation (ASCT), but the number of BV cycles required for an optimal response is unknown. This report describes a patient with relapsed or refractory sALCL who received extended treatment with BV after complete remission before ASCT. This 54‐year‐old man presented with hematuria and abdominal pain in September 2015. Based on the hepatosplenomegaly observed by computerized tomography, the patient was referred to hematology for assessment and was diagnosed with ALK ^−^ sALCL in October 2015. Following unsuccessful first‐line therapy (CHOP + Etoposide), second‐line BV treatment was initiated on 1 February 2016. BV was continued following a suspicion of residual disease in bone marrow after Cycle 4. A recurrence of B symptoms after BV Cycle 8 was judged to be indicative of disease and was completed prior to stem‐cell transplantation. Complete remission has been maintained until the time of writing (May 2017). These observations suggest that extending BV treatment before ASCT can provide benefit, but further clinical studies are needed to determine whether this will improve patient outcomes in sALCL.

Anaplastic large‐cell lymphoma (ALCL), especially anaplastic lymphoma kinase‐negative (ALK^−^) ALCL, is a rare CD30‐expressing aggressive subtype of peripheral T‐cell lymphoma. Brentuximab vedotin (BV; Adcetris^®^ Takeda Pharmaceuticals) is a CD30‐directed antibody–drug conjugate that received FDA approval in August 2011 for the treatment of systemic ALCL (sALCL) after failure of at least one multi‐agent chemotherapy regimen.

BV has shown notable activity in a phase 2 study with an objective response (complete remission [CR] + partial remission [PR]) rate of 86% and a CR rate of 57%; the median duration of objective response and CR was 12.6 and 13.2 months, respectively 1. In a subsequent analysis of patients with relapsed or refractory sALCL, observed for 5 years of follow‐up, the majority of patients achieved clinically significant durable remissions, and a subset may have been cured with BV monotherapy 2.

In contrast, for a young fit patient who has relapsed or who has refractory peripheral T‐cell lymphoma, the standard treatment strategy is to give salvage chemotherapy followed by high‐dose chemotherapy and stem‐cell rescue if the disease is responding to salvage chemotherapy. However, it is not known whether this approach is applicable with BV salvage chemotherapy, where some durable remissions are observed with BV monotherapy alone. In the pivotal trial, no difference in progression‐free survival was observed between patients who received subsequent autologous stem‐cell transplantation (ASCT) and patients who received BV monotherapy without ASCT consolidation 1. Furthermore, it is not known precisely how many BV cycles are optimal before ASCT. In this regard, this article reports on a patient with relapsed or refractory sALCL who received extended treatment with BV after CR before ASCT.

## Case Report

A 54‐year‐old man without any family history of hematologic malignancy was diagnosed with ALK^−^ sALCL in October 2015. He initially presented with hematuria and abdominal pain in September 2015. He had azotemia with an estimated creatinine clearance of 18.8 mL/min/1.73 m^2^. An abdominal computed tomography (CT) scan revealed an approximately 4‐mm‐diameter stone in the right proximal ureter with associated hydronephrosis. In addition, multiple calyceal stones and multifocal decreased perfusion areas were observed in both kidneys. Other findings included a 2‐cm pericardial effusion, hepatosplenomegaly, and an increased attenuation of the spine and pelvic bones. Based on the hepatosplenomegaly observed by CT, the patient was referred to hematology for assessment.

Hematology workup revealed no malignant cutaneous lesions. He had mild B symptoms, including a slightly elevated temperature (38.5°C). Eastern Cooperative Oncology Group (ECOG) performance status was 1. Bone marrow biopsy revealed multiple aggregates of large atypical cells. Immunohistochemical analysis was negative for ALK, CD3, CD8, CD20, and EBER, and positive for CD30. These findings are consistent with a diagnosis of malignant stage 4 ALK^−^ sALCL.

**Figure 1:**
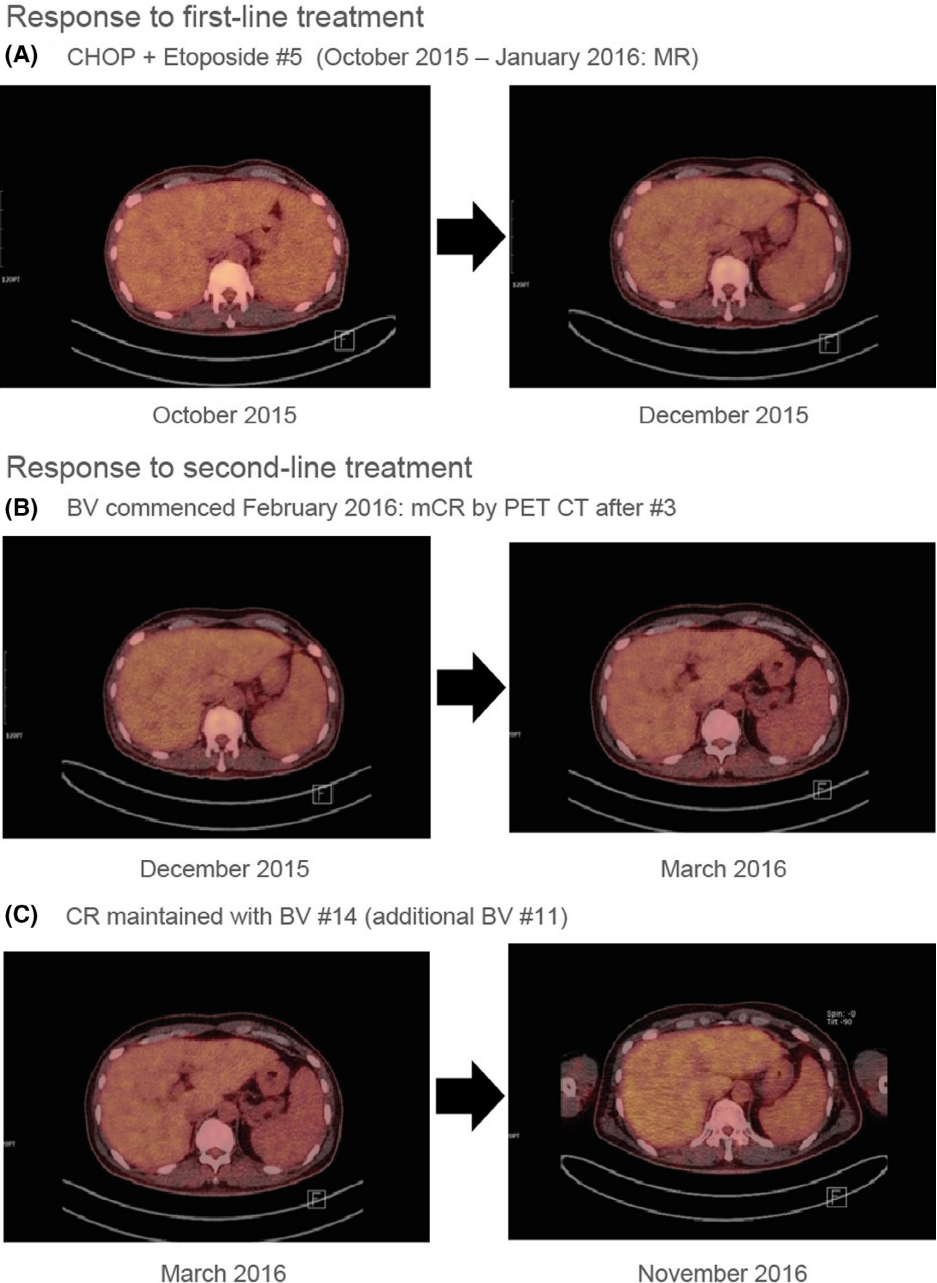
Response to first‐line CHOP + Etoposide was stable disease without significant metabolic change in the liver and spleen. However, after second‐line treatment with BV, metabolic CR was achieved after three cycles. It was maintained after prolonged use of BV.

Based on this diagnosis, first‐line therapy (CHOP + Etoposide) was initiated on 10 October 2016. After 3 months, the patient still complained of abdominal pain, and positron emission tomography–CT (PET‐CT) showed no significant change in diffusely increased F‐18 fluorodeoxyglucose uptake (Fig. 1A). This response was assessed as stable disease, and first‐line therapy was judged to be unsuccessful.

Due to the unsatisfactory cytotoxic response, targeted therapy was preferred, and second‐line BV treatment according to recommended dosing (1.8 mg/kg IV infusion over 30 min every 3 weeks) was initiated on 1 February 2016. After BV Cycle 3, PET‐CT showed metabolic CR and improved hepatosplenomegaly (Fig. 1B), but a suspicion of residual disease was evident in bone marrow after Cycle 4; it is well known that PET‐CT does not reflect BM disease involvement status in T‐cell lymphoma. At Cycle 7, metabolic CR by PET‐CT was sustained and with no evidence in biopsy of bone marrow involvement (no increase in abnormal large cells), indicative of further improvement (late response).

After BV Cycle 8, during a 2‐week stem‐cell collection preparation period for autologous peripheral blood stem‐cell transplantation, the start of BV Cycle 9 was delayed by 1 week, during which time there was a recurrence of B symptoms. Despite the recent negative PET‐CT and bone marrow examinations, the patient still had active disease. Given the history of poor response from CHOP + Etoposide, and the additional late response to BV at Cycle 7, BV was continued until the end of Cycle 14 (Fig. 1C). BV dose was not modified through the 14 cycles.

**Figure 2:**
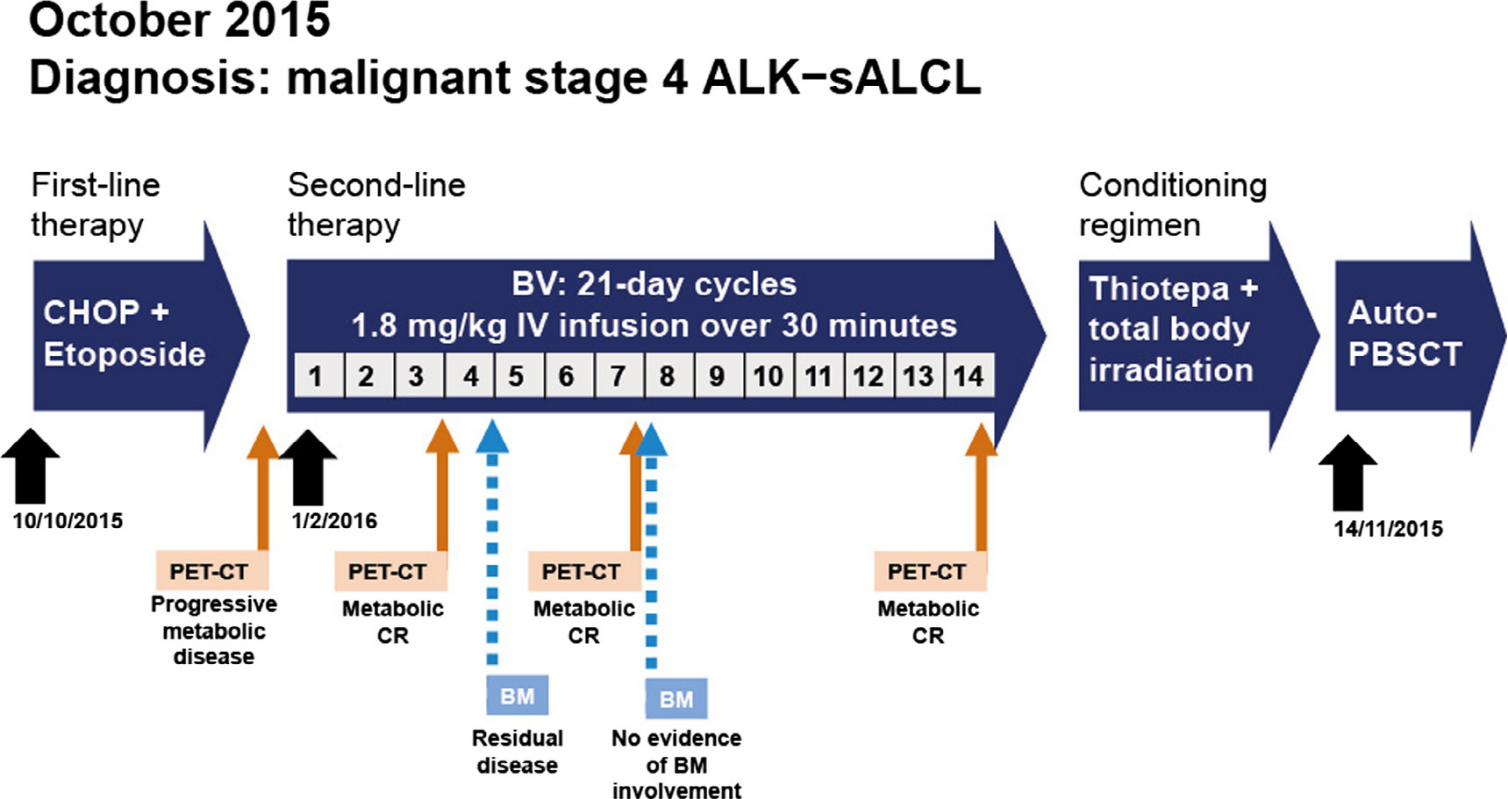
Schematic overview of treatment regimens and monitoring for ALK‐ sALCL.

After BV Cycle 14, a conditioning regimen with the alkylating agent thiotepa and total body irradiation was completed prior to autologous peripheral blood stem‐cell transplantation, which was conducted on 14 November 2016 (Fig. 2). Following transplantation, the patient was considered to be in CR by PET‐CT and that has been maintained until the time of this writing (January 2018; progression‐free survival of BV >24 months).

## Discussion

This case illustrates the clinical benefit of extended BV (14 cycles) in relapsed or refractory sALCL prior to autologous peripheral blood stem‐cell transplantation even after CR.

The optimal treatment duration of BV remains undetermined. In usual salvage chemotherapy before ASCT, 4–6 cycles of salvage chemotherapy are performed. However, BV does not share its anticancer mechanism of action with conventional cytotoxic chemotherapy regimens and is tolerable with extended use 3. Moreover, the number of BV cycles required for best response in sALCL has not yet been established.

The situation is more complicated if BV is the first drug to produce an objective response in chemorefractory peripheral T‐cell lymphoma, and there is considerable debate regarding the optimal duration of salvage BV therapy before ASCT. A previous patient of ours with chemorefractory sALCL given ASCT following a CR after four cycles of BV salvage chemotherapy had a very short progression‐free survival of 3 months. Based on this experience, we have tried to extend BV salvage chemotherapy before ASCT in patients who are chemorefractory to previous cytotoxic chemotherapy.

## Conclusion

In conclusion, it is hoped that this clinical experience will help practitioners to consider extended BV treatment before ASCT, especially in chemorefractory disease. This case illustrates that further benefit may be expected from prolonged use of BV before ASCT in sALCL, but practical experience on the optimal BV duration of treatment in relapsed or refractory sALCL is insufficient. Further clinical studies are required to determine whether extended treatment with BV will eradicate the residual disease that may persist before ASCT, thus improving outcomes for patients with this life‐threatening disease.

## Authorship

YK: is the consulting physician involved in the patient's care, conceived of the case report, critically reviewed all stages of the manuscript development, got approved the final version for submission to the *European Journal of Haematology*, and took full responsibility for the contents of this publication.

## Conflict of Interest

Dr Koh has no conflict of interest relevant to this report.
